# Methemoglobinemia: A Life-threatening Complication of Topical Pharyngeal Anesthetics

**DOI:** 10.7759/cureus.7900

**Published:** 2020-04-30

**Authors:** Sana Riaz, Pujitha Kudaravalli, Sheikh A Saleem, David Heisig

**Affiliations:** 1 Internal Medicine, State University of New York (SUNY) Upstate Medical University, Syracuse, USA; 2 Gastroenterology, State University of New York (SUNY) Upstate Medical University, Syracuse, USA

**Keywords:** methemoglobinemia, lidocaine, topical anesthesia

## Abstract

Drug-induced methemoglobinemia can be caused due to topical anesthetics, dapsone, nitrates (e.g., nitroglycerin), and metoclopramide. Cyanosis in the setting of topical anesthetic use, along with the arterial blood gas results indicating hypoxemia, points towards the diagnosis of methemoglobinemia. We highlight the potential complication with the use of topical pharyngeal benzocaine through this case presentation.

## Introduction

Topical pharyngeal anesthetics such as benzocaine are used in conjunction with moderate sedation to increase patient comfort during esophagogastroduodenoscopy (EGD). Topical pharyngeal anesthetics can cause methemoglobinemia, which is a rare and life-threatening complication [1]. We present a case of a 22-year-old healthy female with no known co-morbidities who developed symptoms of shortness of breath following the use of topical anesthesia for EGD and was subsequently diagnosed with methemoglobinemia based on her arterial blood gas and methemoglobin levels. Through this case, we want to highlight the possible complication of methemoglobinemia with the use of benzocaine.

## Case presentation

A 22-year-old female with no prior medical history presented with odynophagia after swallowing a piece of meat. She underwent inpatient EGD and received topical pharyngeal benzocaine spray along with moderate sedation. EGD was unremarkable, but during recovery, the patient became dyspneic, cyanotic, and her oxygen saturation (SaO2) dropped to 85% on room air. On chest examination, she had no wheezing, rhonchi, or crackles. Arterial blood gas showed pH 7.50, partial pressure of carbon dioxide 21 mmHg, partial pressure of oxygen 409 mmHg, and SaO2 99%. Blood work showed a methemoglobin level of 27.8%. She was transferred to the intensive care unit (ICU) and was given supplemental oxygen through facemask and IV ascorbic acid, which resolved her cyanosis and hypoxemia. Repeat methemoglobin level was 8.3% with normal oxygen saturation on room air. Upon stabilization, she was discharged home 48 hours after her initial presentation.

## Discussion

Methemoglobinemia is the oxidation of ferrous to ferric iron within hemoglobin, impairing its ability to transport oxygen, leading to tissue hypoxemia and death [2]. The etiologies of methemoglobinemia are hemoglobinopathy (hemoglobin M), hereditary nicotinamide adenine dinucleotide reductase (NADH) enzyme deficiency, and drug induced [3]. The diagnosis of clinical methemoglobinemia depends on the co-oximeter measurement of an elevated methemoglobin level (>1% or 2%) [4]. Treatments include the use of methylene blue and other agents such as ascorbic acid and riboflavin. 

Drug-induced methemoglobinemia is commonly seen in patients undergoing endoscopic procedures such as EGDs, laryngoscopies, and bronchoscopies. It should be considered in patients presenting with cyanosis and hypoxemia following topical pharyngeal anesthetic use. Despite the rare incidence, most cases of topical-anesthesia-induced methemoglobinemia are secondary to benzocaine. Benzocaine is considered to be a more powerful oxidizing agent compared to lidocaine, and a dose-response relationship has been observed between benzocaine and methemoglobin [5]. There is insufficient evidence for methemoglobinemia caused by topical pharyngeal lidocaine alone and reported cases of lidocaine associated methemoglobinemia occurred in patients with either cardiorespiratory diseases or with the use of other anesthetic agents [5]. In a review of 242 cases of methemoglobinemia by Guay et al., benzocaine was implicated in 66% of all cases, while lidocaine accounted for only about 5% [4]. More recently, in a retrospective study by Vallurupalli et al. to determine the incidence of methemoglobinemia with the use of different local anesthetics, it was noted that all cases resulted from the use of 20% benzocaine spray. No cases were reported with lidocaine spray [6]. This case has been presented as an abstract to the American Journal of Gastroenterology [7]. (Abstract: Riaz S, Kudaravalli P, Saleem SA, Heisig D. Methemoglobinemia: A Life-Threatening Complication of Topical Pharyngeal Anesthetics. American Journal of Gastroenterology; October 2019).

## Conclusions

Methemoglobinemia is a rare complication in clinical practice; however, it is commonly seen in patients undergoing endoscopic procedures. Methemoglobinemia is believed to be seen in patients with genetic predispositions; however, the increasing use of topical anesthetics during procedures has made methemoglobinemia a disease entity that every clinical provider should be able to recognize. Drug-induced methemoglobinemia is more commonly seen with benzocaine than lidocaine. Therefore, if the use of topical pharyngeal anesthetics is deemed necessary during an endoscopic procedure, topical viscous lidocaine should be preferred over benzocaine to avoid this potentially life-threatening complication.

## References

[1] Filipiak-Strzecka D, Kasprzak JD, Wiszniewska M, Walusiak-Skorupa J, Lipiec P (2015). The influence of lidocaine topical anesthesia during transesophageal echocardiography on blood methemoglobin level and risk of methemoglobinemia. Int J Cardiovasc Imaging.

[2] Wright RO, Lewander WJ, Woolf AD (1999). Methemoglobinemia: etiology, pharmacology, and clinical management. Ann Emerg Med.

[3] Weiss LD, Generalovich T, Heller MB, Paris PM, Stewart RD, Kaplan RM, Thompson DR (1987). Methemoglobin levels following intravenous lidocaine administration. Ann Emerg Med.

[4] Guay J (2009). Methemoglobinemia related to local anesthetics: a summary of 242 episodes. Anesth Analg.

[5] Karim A, Ahmed S, Siddiqui R, Mattana J (2001). Methemoglobinemia complicating topical lidocaine used during endoscopic procedures. Am J Med.

[6] Vallurupalli S, Manchanda S (2011). Risk of acquired methemoglobinemia with different topical anesthetics during endoscopic procedures. Local Reg Anesth.

[7] Riaz S, Kudaravalli P, Saleem SA, Heisig D (2019). Methemoglobinemia: a life-threatening complication of topical pharyngeal anesthetics. Am J Gastroenterol.

